# Notfallsituationen und Krankenhauszuweisungen in Pflegeeinrichtungen – ein Scoping-Review zu Begleitumständen und versorgungsrelevanten Maßnahmen

**DOI:** 10.1007/s00103-022-03543-w

**Published:** 2022-05-17

**Authors:** Carsten Bretschneider, Juliane Poeck, Antje Freytag, Andreas Günther, Nils Schneider, Sven Schwabe, Jutta Bleidorn

**Affiliations:** 1 Institut für Allgemeinmedizin, Universitätsklinikum Jena, Bachstr. 18, 07743 Jena, Deutschland; 2 Fachbereich Feuerwehr, Braunschweig, Deutschland; 3 Institut für Allgemeinmedizin und Palliativmedizin, Medizinische Hochschule Hannover, Hannover, Deutschland

**Keywords:** Notfall, Pflegeheim, Hospitalisierung, Interventionen, Geriatrie, Emergency, Nursing home, Hospitalization, Interventions, Geriatrics

## Abstract

**Hintergrund:**

Pflegeheimbewohner:innen werden häufiger hospitalisiert als nicht institutionalisierte Gleichaltrige. Eine Vielzahl an Rettungsdiensteinsätzen und Krankenhauszuweisungen wird als potenziell vermeidbar eingeordnet.

**Ziele der Arbeit:**

Zuweisungsbegünstigende Begleitumstände bei Notfallsituationen in Pflegeeinrichtungen sowie Ansätze zur Reduktion von Krankenhauszuweisungen sollen identifiziert werden, um die Komplexität der Versorgungsprozesse und Handlungsperspektiven aufzuzeigen.

**Material und Methoden:**

Scoping-Review mit Analyse aktueller Original- und Übersichtsarbeiten (2015–2020) in den Datenbanken PubMed, CINAHL sowie per Handsuche.

**Ergebnisse:**

Aus 2486 identifizierten Studien wurden 302 Studien eingeschlossen. Verletzungen, Frakturen, kardiovaskuläre und respiratorische Erkrankungen sowie Infektionskrankheiten sind die häufigsten retrospektiv erfassten Diagnosegruppen. Hinsichtlich der einweisungsbegünstigenden Umstände konnten verschiedene Aspekte identifiziert werden: bewohnerbezogene (z. B. Multimorbidität, fehlende Patientenverfügungen), einrichtungsbezogene (u. a. Personalfluktuation, Unsicherheiten), arztbezogene (z. B. mangelnde Erreichbarkeit, erschwerter Zugang zu Fachärzt:innen) und systembedingte (z. B. eingeschränkte Möglichkeiten zur Diagnostik und Behandlung in Einrichtungen). Verschiedene Ansätze zur Verminderung von Krankenhauszuweisungen sind in Erprobung.

**Diskussion:**

Vielfältige Begleitumstände beeinflussen das Vorgehen in Notfallsituationen in Pflegeeinrichtungen. Interventionen zur Reduktion von Krankenhauszuweisungen adressieren daher u. a. die Stärkung der Kompetenz des Pflegepersonals, die interprofessionelle Kommunikation und systemische Ansätze. Ein umfassendes Verständnis der komplexen Versorgungsprozesse ist die wesentliche Grundlage für die Entwicklung und Implementierung effektiver Interventionen.

**Zusatzmaterial online:**

Zusätzliche Informationen sind in der Online-Version dieses Artikels (10.1007/s00103-022-03543-w) enthalten.

## Abstract (en)

**Background:**

Nursing home residents are more likely to be hospitalized as non-institutionalized peers. A large number of emergency medical services (EMS) and emergency department visits are classified as potentially avoidable.

**Objectives:**

To identify circumstances that increase the number of emergency situations in nursing homes and approaches to reduce hospital admissions in order to illustrate the complexity and opportunities for action.

**Materials and methods:**

Scoping review with analysis of current original and peer-reviewed papers (2015–2020) in PubMed, CINAHL, and hand-search databases.

**Results:**

From 2486 identified studies, 302 studies were included. Injuries, fractures, cardiovascular, respiratory, and infectious diseases are the most frequent diagnostic groups that have been retrospectively recorded. Different aspects could be identified as circumstances inducing emergency department visits: resident-related (e.g., multimorbidity, lack of volition, and advance directives), facility-related (e.g., staff turnover, uncertainties), physician-related (lack of accessibility, challenging access to specialists), and system-related circumstances (e.g., limited possibilities for diagnostics and treatment in facilities). Multiple approaches to reduce emergency department visits are being explored.

**Conclusions:**

A variety of circumstances influence the course of action in emergency situations in nursing facilities. Therefore, interventions to reduce emergency department visits address, among other things, strengthening the competence of nursing staff, interprofessional communication, and systemic approaches. A comprehensive understanding of the complex processes of care is essential for developing and implementing effective interventions.

## Einleitung

In Deutschland werden ca. 3,4 Mio. Menschen als pflegebedürftig eingestuft, von denen 818.000 in Einrichtungen der stationären Langzeitpflege, im Folgenden als Pflegeeinrichtungen bezeichnet, versorgt werden [1]. Dabei stellt die steigende Zahl an multimorbiden Bewohner:innen die Einrichtungen vor zunehmende Herausforderungen [2]. Insbesondere Notfallsituationen, hier verstanden als physische oder psychische Veränderungen des Gesundheitszustands, für welche die Patientin/der Patient oder Dritte eine unverzügliche Versorgung erachten [3], führen bei Pflegeheimbewohner:innen häufig zu Rettungsdiensteinsätzen und Krankenhauszuweisungen [4]. Pro Jahr werden 29–62 % der Pflegeheimbewohner:innen mindestens einmal in der Notaufnahme vorstellig [5]. Sie werden häufiger hospitalisiert als nicht institutionalisierte Gleichaltrige [6]. Die Inanspruchnahme ambulanter Notfallversorgungen steigt im Folgejahr der Einweisung in eine Pflegeeinrichtung [7].

Rettungsdiensttransporte und Krankenhauszuweisungen können mit physischen und psychischen Funktionseinbußen sowie gesundheitlichen Risiken einhergehen [8], sog. Hazards of Hospitalization [9]. Zudem überfordert der Kontextwechsel häufig die Bewältigungsstrategien der Bewohner:innen (Relocation Stress; [10, 11]). 4–55 % der Krankenhauszuweisungen aus Pflegeeinrichtungen werden als potenziell vermeidbar eingeschätzt [12], wobei Vermeidbarkeit nicht definiert bzw. operationalisiert wird.

In den letzten Jahren sind u. a. in systematischen Reviews verschiedene Einflussfaktoren von Hospitalisierungen und Transferentscheidungen in Pflegeeinrichtungen untersucht worden [12–15]. Zudem werden in bisherigen Studien einzelne Umstände beleuchtet, die eine wesentliche Rolle bei der Entscheidung für oder gegen eine Krankenhauszuweisung spielen und z. T. bedeutender als die klinische Präsentation sind. Hierzu gehören bspw. Mangel an Pflegepersonal, eingeschränkte Erreichbarkeit ärztlicher Ansprechpartner:innen und fehlende Patientenverfügungen [16, 17]. Zum Thema Notfallsituationen in Pflegeeinrichtungen bestehen aber auch einige Forschungslücken (siehe Infobox 1).

Das vorliegende Scoping-Review hat zum Ziel, einen Überblick über die Verknüpfung von Notfallsituationen, Begleitumständen und möglichen Lösungsansätzen zu geben und ein tieferes Verständnis für den komplexen Versorgungsprozess in Notfallsituationen zu schaffen. Es werden häufige medizinische Notfallsituationen in Pflegeeinrichtungen skizziert und Begleitumstände identifiziert, die die Wahrscheinlichkeit einer Krankenhauszuweisung erhöhen können. Ansätze und Maßnahmen zur Reduktion der Zuweisungen aus Pflegeeinrichtungen werden integriert.

## Methode

Um einen Überblick über Notfallsituationen und Krankenhauszuweisungen von Pflegeheimbewohner:innen zu schaffen, führten die Autor:innen ein Scoping-Review in Anlehnung an Arksey und O’Malley (2005) durch, mit einigen Erweiterungen nach Peters et al. (2020; [18, 19]). Ein Studienprotokoll wurde verfasst.

### Suchstrategie

Die Suche aktueller Original- und Übersichtsarbeiten erfolgte in den Datenbanken PubMed und CINAHL für den Zeitraum vom 01.01.2015–31.12.2020 mit den Stichworten u. a. „nursing home“, „emergencies“, „patient transfer“, „hospital admission“, „emergency medical services“. Die Suchsyntax befindet sich im Onlinematerial zu diesem Beitrag. Ergänzend wurden Maßnahmen zur Vermeidung von Krankenhauseinweisungen manuell in Google und Google Scholar recherchiert. Zudem wurden die aktuell geförderten Innovationsfondsprojekte bezüglich der Einschlusskriterien sowie die Literaturverzeichnisse der ausgewählten Studien gescreent.

### Ein- und Ausschlusskriterien

Die Einschlusskriterien sind entsprechend des P(opulation)-C(onzept)-C(ontext)-Schemas dargestellt [20].

- *Population:* Bewohner:innen in Einrichtungen der stationären Langzeitpflege
- *Concept: *Notfallsituationen in Pflegeheimen, die zu Krankenhauseinweisungen führen; Ursachen von Notfallsituationen sowie Maßnahmen zur Reduktion von Krankenhauszuweisungen
- *Context: *deutsch- und englischsprachige Studien, die zwischen 2015 und 2020 publiziert worden sind (alle Studientypen). Ergebnisse von systematischen Reviews werden unabhängig vom Erscheinungsjahr der Primärliteratur eingeschlossen.

Ausschlusskriterien:

- Studien zu geplanten Krankenhauszuweisungen
- Rehabilitative Behandlungen bei Bewohner:innen in stationärer Langzeitpflege (Skilled Nursing Facilities)
- Wiederaufnahme in ein Krankenhaus aus einer stationären Pflegeeinrichtung innerhalb von 30 Tagen nach der initialen Entlassung aus dem Krankenhaus
- Krankenhauszuweisungen im Rahmen der SARS-CoV-2-Pandemie

Die Kriterien zur Studienauswahl und Datenextraktion wurden im Forschungsteam konsentiert. Die Datenextraktion erfolgte durch den Autor CB anhand deduktiv abgeleiteter Kategorien, deren Unterkategorien induktiv ergänzt wurden, anhand folgender Bereiche: *Notfallsituationen* (Diagnosen, Symptome/Symptomkomplexe), *einweisungsbegünstigende Begleitumstände* (bewohnerbezogen, einrichtungsbezogen, arztbezogen, systembedingt) und *Maßnahmen zur Vermeidung von Einweisungen* (z. B. Heimarztkonzepte, interdisziplinäre Teams, Edukation der Pflegekräfte, Tool-basierte Interventionen, Telemedizin, mobile Radiologiesysteme, vorausschauende gesundheitliche Versorgungsplanung (Advance Care Planning, ACP) und End of Life Care). Bei auftretenden Unsicherheiten wurde der Einschluss der Studien im Forscherteam diskutiert.

## Ergebnisse

**Figure 1:**
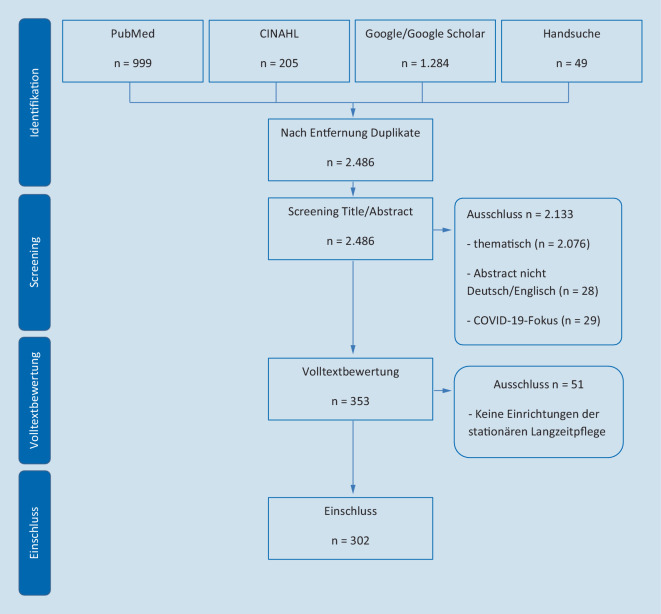

Insgesamt wurden aus 2486 identifizierten Studien 302 in das Scoping-Review eingeschlossen (Abb. 1). Von den 302 eingeschlossenen Studien befassen sich 45 Studien primär mit Notfallsituationen, 102 Studien mit Begleitumständen und 162 Studien mit Interventionen oder Maßnahmen. Dabei stammen 107 Studien aus den USA und Kanada, 147 Studien aus Europa, darunter 78 aus Deutschland. Weiterhin wurden 41 Studien aus Australien und Neuseeland sowie 7 Studien aus Asien eingeschlossen. Nachfolgend wird die Synthese aus primär ausgewählten, versorgungsrelevanten Ergebnissen dargestellt.

### Überblick zu Notfallsituationen

In Studien werden Notfallsituationen vorwiegend retrospektiv anhand von Abrechnungsdaten, Entlassungsberichten oder Rettungsdienstprotokollen analysiert. Die häufigsten nach einer Krankenhauszuweisung erhobenen Diagnosegruppen stellen Weichteilverletzungen und Frakturen, kardiovaskuläre und respiratorische Erkrankungen sowie Infektionskrankheiten dar [21–23]. Dabei sind Pneumonie, Herzinsuffizienz, Sepsis, Demenz und Harnwegsinfekt häufige Einzeldiagnosen [21, 22]. Sturzbedingte Zuweisungen tragen erheblich zu Krankenhauszuweisungen bei [12, 21, 24]. Weitaus seltener sind Untersuchungen, die auf vom Pflegepersonal identifizierten Symptomen einer Notfallsituation basieren. Hier werden am häufigsten allgemeines Unwohlsein, Atemnot, Veränderungen im Bewusstseinszustand, Fieber, Schmerzen, verminderte Nahrungs- oder Flüssigkeitsaufnahme und abnormale Vitalparameter genannt [25].

### Zuweisungsbegünstigende Begleitumstände

**Figure 2:**
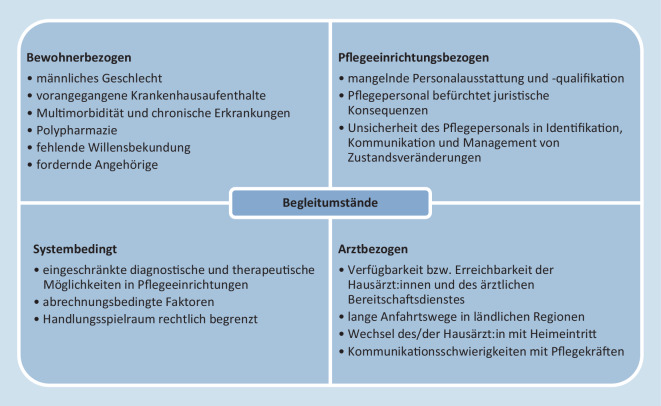

Die identifizierten zuweisungsbegünstigenden Begleitumstände wurden in bewohnerbezogene, pflegeeinrichtungsbezogene, arztbezogene und systembedingte Begleitumstände unterteilt (Abb. 2). Zu bewohnerbezogenen Begleitumständen können Charakteristika und epidemiologische Faktoren des/der Bewohner:in (z. B. Alter, Geschlecht, Vorerkrankungen und Medikation) zugeordnet werden. Als pflegeeinrichtungsbezogene Begleitumstände werden v. a. organisatorische und personelle Einflüsse innerhalb der Pflegeeinrichtung subsumiert. Welchen Einfluss die Ressource ärztliches Personal auf Einweisungsentscheidungen hat, wird unter arztbezogenen Umständen zusammengefasst. Unter den systembedingten Begleitumständen sind Einflüsse von Versorgungs- und Vergütungsstrukturen dargestellt.

#### Bewohnerbezogene Begleitumstände

Einige Studien weisen darauf hin, dass häufiger männliche als weibliche Pflegeheimbewohner:innen hospitalisiert werden [15, 26, 27]. Welchen Einfluss das Alter der Bewohner:innen auf die Häufigkeit von Krankenhauszuweisungen hat, kann nicht eindeutig belegt werden [5, 26]. Vorangegangene Hospitalisierungen und wiederholte Notaufnahmevorstellungen können die Häufigkeit erneuter Krankenhauszuweisungen [28–30] erhöhen ebenso wie ein Wechsel der Umgebung. Insbesondere in den ersten 3 Monaten nach Aufnahme in eine Pflegeeinrichtung sind Einweisungen häufiger [7, 15, 26]. Multimorbidität [28, 29], chronische Erkrankungen wie chronisch obstruktive Lungenerkrankungen, Asthma und Herzinsuffizienz [15], onkologische Erkrankungen [31] und kognitive Einschränkungen [32] erhöhen ebenso wie zunehmende Pflegebedürftigkeit [27], Schluckstörungen und bestimmte Interventionen (z. B. Anlage einer PEG-Sonde) das Risiko für Notfallsituationen mit nachfolgender Krankenhauszuweisung [15]. Auch Polypharmazie scheint mit einem höheren Risiko für Krankenhauszuweisungen assoziiert zu sein [22, 27, 33].

Angehörige nehmen in Transferentscheidungen eine wichtige Rolle ein, sie werden in einigen Untersuchungen vom Pflegepersonal als zuweisungsbegünstigend angesehen [25, 34, 35]. Ihr Einfluss auf Transferentscheidungen variiert stark, Konflikte mit Behandler:innen entstehen bei der Umsetzung des Bewohnerwillens [36]. Angehörige sehen in Hospitalisierungen häufig die Chance, den Gesundheitszustand der Bewohner:innen zu verbessern [37], ohne sich dabei der Risiken einer stationären Behandlung bewusst zu sein [12]. Zustandsveränderungen der Bewohner:innen können bei Angehörigen Ängste und Unsicherheiten auslösen, weshalb eine Aufklärung über Prognose und Nutzen einer Hospitalisierung wichtig ist [34]. Eine frühzeitige Einbindung und klare Kommunikation zwischen Angehörigen und Pflegepersonal über Bewohnerpräferenzen können zur besseren Versorgung am Lebensende beitragen [14, 25]. Ebenso wichtig erscheinen eindeutig formulierte Patientenverfügungen [38].

#### Pflegeeinrichtungsbezogene Begleitumstände

Bekannt ist, dass der Mangel an personeller Ausstattung und die eingeschränkte Verfügbarkeit von qualifiziertem Pflegepersonal die Versorgungsgüte in Pflegeeinrichtungen reduzieren und Krankenhauszuweisungen begünstigen können [35, 38]. Ebenso beeinflussen Unsicherheiten der Pflegekräfte bei der Identifikation, Kommunikation und dem Management von Zustandsveränderungen den Umgang mit Notfallsituationen und werden daher als Verbesserungsmöglichkeit benannt [14, 25]. Von primärversorgenden Ärzt:innen wird unzureichend ausgebildetes Pflegepersonal als Risikofaktor für Hospitalisierungen eingeschätzt, insbesondere außerhalb der hausärztlichen Erreichbarkeit [11]. Daneben schätzt das Pflegepersonal die Dringlichkeit für einen Arztbesuch zum Teil höher ein als der/die behandelnde Hausärzt:in [39]. Kommt es zu einer zeitlichen Verzögerung oder bleibt der Hausbesuch gänzlich aus, wird in der Regel der Rettungsdienst gerufen. Das unterstreicht den Stellenwert der interprofessionellen Kommunikation zwischen den Vertreter:innen der beteiligten Gesundheitsberufe, was wiederholt als Schwierigkeit identifiziert wurde und Ansätze für wirksame Interventionen bietet [12, 38]. Als bedrohlich wahrgenommene, mögliche juristische Konsequenzen und der Wunsch nach Absicherung werden vom Pflegepersonal als weitere Gründe für Krankenhauszuweisungen angeführt [13, 14].

ACP kann dabei unterstützen, Krankenhauszuweisungen zu verringern [40]. Sind Pflegeziele und das Vorgehen bei Zustandsveränderungen vorab geklärt, erleichtert dies das Vorgehen in Notfallsituationen [41]. Eine zeitnahe palliative Anbindung des/der Bewohner:in z. B. durch frühzeitige Konsultationen, Kommunikation mit palliativmedizinisch weitergebildeten Pflegekräften und verbesserte Symptomkontrolle kann die Häufigkeit von Krankenhauszuweisungen verringern [42, 43].

#### Arztbezogene Begleitumstände

In mehreren Studien wird die mangelnde Erreichbarkeit der primärversorgenden Ärzt:innen bei Eintreten der Notfallsituation als relevante Ursache für Krankenhauszuweisungen identifiziert [12, 38, 44, 45]. In ländlichen Gebieten sind die Anfahrtswege für den ärztlichen Bereitschaftsdienst länger, sodass in einigen Fällen zur Verkürzung der Wartezeiten eher der Rettungsdienst alarmiert wird [45]. Angaben zur Häufigkeit von direkten Hausarztkontakten kurz vor Hospitalisierung schwanken zwischen 11 % und 44 % [15, 25]. Dabei könnten insbesondere Bewohner:innen am Lebensende von solchen Konsultationen profitieren, da der/die Hausärzt:in im Vergleich zu Rettungsdienst und ärztlichem Bereitschaftsdienst häufiger Maßnahmen veranlasst, die den Verbleib in der gewohnten Umgebung ermöglichen [46]. Weniger Krankenhauszuweisungen können bei Bewohner:innen auftreten, die mit dem Eintritt in die Pflegeeinrichtung die ursprünglich primärversorgenden Ärzt:innen behalten [47].

#### Systembedingte Begleitumstände

Diagnostische und therapeutische Möglichkeiten zur medizinischen Vor-Ort-Versorgung in Pflegeeinrichtungen bestehen bisher nur in geringem Umfang. So sind bspw. radiologische Diagnostik und intravenöse Medikamentengabe in der Regel nicht möglich [9, 30], Hospitalisierungen können die Folge sein.

Zudem sind in einigen Bundesländern systembedingte Anreize für Transport (zum Krankenhaus) statt Versorgung vor Ort zu verzeichnen: Rettungsdiensteinsätze ohne Transport werden dort nicht regelhaft vergütet. Auch die Finanzierung bzw. Trägerschaft von Einrichtungen kann Hospitalisierungen beeinflussen. In einem städtischen Versorgungsbereich Deutschlands waren Notfallrettungseinsätze in Einrichtungen mit privat-überregionaler Trägerschaft häufiger [4]. Zudem weisen privat geführte Pflegeeinrichtungen in Untersuchungen aus den USA höhere Zuweisungsraten auf. Mögliche Ursachen sind bspw. eine spezielle Versichertenstruktur und insgesamt niedrigere organisationale, personelle und räumliche Ressourcen [15, 47].

### Maßnahmen

National und international werden vielfältige Maßnahmen zur Reduzierung von Krankenhauszuweisungen bei Pflegeheimbewohner:innen und zur Verbesserung der Versorgungsqualität erprobt. Zentrale und Erfolg versprechende Ansätze bestehen z. B. in:

- Tools zur zeitnahen Erkennung und Kommunikation von Zustandsveränderungen und klaren Anweisungen für Notfallsituationen [48],
- der Verfügbarkeit ärztlicher Versorgung, vor allem außerhalb der Sprechzeiten, bspw. über telemedizinische Anwendungen [49] oder mobile Versorgungsteams [48],
- der Förderung der Vernetzung und interdisziplinären Zusammenarbeit, bspw. über regelmäßige Heimvisiten [49] sowie durch hausärztlich geriatrische pharmakologische Teams [50]. In Deutschland wird dieser Ansatz über die Heim-Kooperationsverträge (entsprechend der Vereinbarung nach § 119b Abs. 2 Fünftes Buch Sozialgesetzbuch (SGB V) gefördert [51]);
- Zugang zu radiologisch-diagnostischen Möglichkeiten verbessern [52, 53],
- edukative Maßnahmen für Pflege- und ärztliches Personal zu Dokumentation, interprofessioneller Kooperation und Kommunikation [50, 54],
- intensivierte Kommunikation zwischen Betroffenen, Angehörigen, Pflegenden und Hausärzt:innen zur Umsetzung der Patientenbedürfnisse, z. B. im Rahmen des Wiesbadener Palliativpasses [55],
- Unterstützung der Pflegeeinrichtungen durch spezialisierte Pflegekräfte, welches sich v. a. im amerikanischen Raum etabliert hat [56, 57],
- Schaffung ökonomischer Anreize, z. B. Zusatzentgelte für aufwendigere Versorgung in der Pflegeeinrichtung anstatt einer Einweisung [57, 58],
- Etablierung von (geringen) Einweisungsraten als Qualitätsparameter [59],
- Umsetzung vorausschauender Versorgungsplanung (Advance Care Planning, ACP) mit entsprechend geschulten Gesprächsbegleiter:innen [60].

In Deutschland werden durch den Innovationsfonds des Gemeinsamen Bundesausschusses aktuell verschiedene Projekte gefördert, die die Versorgungsoptimierung in Pflegeeinrichtungen zum Ziel haben (Tab. 1).

**Table 1:** 

Projekte Innovationsfonds	Beschreibung	Status
*BEVOR* *(Patientenrelevante Auswirkungen von Behandlung im Voraus planen)*	Konzept zur strukturierten Vorausplanung; Bewohner:innen und deren Angehörige können Behandlungswünsche mit Unterstützung durch qualifizierte Gesprächsbegleiter:innen und Hausärzt:innen im Voraus festlegen	Laufend
*Co-Care* *(Coordinated Medical Care)*	Erweiterte koordinierte ärztliche Pflegeheimversorgung: Optimierung der Schnittstelle zwischen Pflegepersonal und Ärzt:innen durch gemeinsame haus- und fachärztliche Visiten, Bildung von Ärzteteams, gemeinsame elektronische Patientenakte, erweiterte Erreichbarkeit der Ärzt:innen	Abschlussbericht wird erstellt
*Comm4Care SAN* *(Optimierung der interprofessionellen Kommunikation)*	Stärkung der interprofessionellen Vernetzung mithilfe einer digitalen Kommunikationsplattform zwischen Ärzt:innen, Pflegekräften und Bewohner:innen	Laufend
*Interprof ACT* *(Verbesserung ärztlich-pflegerischer Zusammenarbeit)*	Maßnahmenpaket zur Verbesserung der Zusammenarbeit und Kommunikation von Pflegekräften in Pflegeeinrichtungen und Hausarztpraxen	Abschlussbericht wird erstellt
*HOMERN* *(Hospitalisierung und Notaufnahmebesuche von Pflegeheimbewohner:innen)*	Erfassung von Behandlungsanlässen in Notaufnahmen und Krankenhäusern sowie vorausgegangenen Akutereignissen und beteiligten Professionen	Projekt beendet
*MVP-STAT (Bedarfsgerechtigkeit der medizinischen Versorgung Pflegeheimbewohner:innen)*	Umfassende Analysen zur Identifikation von Barrieren und Förderfaktoren der medizinischen Versorgung, fachärztlichen Versorgung pflegebedürftiger Heimbewohner:innen, Entwicklung partizipativer Strategien	Projekt beendet
*Novelle* *(Sektorenübergreifendes & integriertes Notfall- und Verfügungsmanagement in stationärer Langzeitpflege)*	Entwicklung interdisziplinärer Handlungsempfehlungen für Notfallsituationen, um die Handlungs- und Rechtssicherheit von Pflegefachpersonen zu verbessern und Krankenhauszuweisungen zu reduzieren	Laufend
*Optimal@NRW* *(Optimierte Akutversorgung geriatrischer Patienten durch ein intersektorales telemedizinisches Kooperationsnetzwerk)*	Optimierung der intersektoralen Akutversorgung geriatrischer Patient:innen durch Frühwarnsysteme, Telekonsultationssysteme und sektorenübergreifende digitale Behandlungsdokumentation	Laufend
*PSK* *(Bedarfsgerechte Versorgung von Pflegeheimbewohner:innen durch Reduktion Pflegeheim-sensitiver Krankenhausfälle)*	Erstellung eines Katalogs über vermeidbare Krankenhausbehandlungen für Pflegeheimbewohner:innen; Erarbeitung von Strategien zur Vermeidung unnötiger Krankenhausbehandlungen	Abschlussbericht wird erstellt
*SaarPHIR* *(Saarländische Pflegeheimversorgung Integriert Regelhaft)*	Verbesserung der ärztlichen Verfügbarkeit in Pflegeeinrichtungen durch regionale Versorgerteams aus Haus- und Fachärzt:innen; geschulte Pflegekräfte als Ansprechpartner:innen; für die Ärzt:innen interprofessionelle Team- und Fallbesprechungen; gemeinsame Versorgungsplanung	Abschlussbericht wird erstellt

^a^Gemeinsamer Bundesausschuss Innovationsfonds: Förderprojekte. https://innovationsfonds.g-ba.de/projekte/ (Zugegriffen 15.04.2022)

## Diskussion

Notfallsituationen und Krankenhauszuweisungen bei Pflegeheimbewohner:innen sind nicht nur durch die medizinische Situation, sondern auch durch vielfältige Begleitumstände gekennzeichnet, wobei bewohnerbezogene, pflegeeinrichtungsbezogene, arztbezogene und systembedingte Begleitumstände unterschieden werden können. Diese Umstände stellen ein komplexes System dar und beeinflussen sowohl das Risiko für das Auftreten einer Notfallsituation und die jeweilige Vorgehensweise als auch Entscheidungen für oder gegen eine Krankenhauseinweisung.

In der Wahrnehmung und Einschätzung von Notfallsituationen durch die beteiligten Pflegekräfte nehmen die Begleitumstände eine zentrale Rolle ein – im Gegensatz zu ärztlichem Personal, bei dem die medizinischen Aspekte im Vordergrund stehen [61]. Um Pflegefachkräfte besser auf Notfallsituationen vorzubereiten und Fähigkeiten bzgl. Dokumentation und Kommunikation zu stärken, wurden zahlreiche Programme zur Weiterbildung von Pflegekräften entwickelt [62]. Gerade Pflegefachkräfte finden sich allerdings häufig in Rollenkonflikten – als Vertraute der Bewohner:innen, als Ansprechpartner:innen für Angehörige und als Fachpersonal, das mit Rettungsdienst und Ärzt:innen interagiert [13, 63]. Daher erscheint es relevant, entsprechende Interventionen auch auf begleitende Umstände, weitere agierende Personen und den prozessualen Rahmen einer Entscheidungsfindung auszurichten.

Vielfältige Maßnahmen sind in Erprobung, die unterschiedlich breit ansetzen: Mit der „Mobilen Radiologie“ [52] wird bspw. gezielt das Problem der häufigen Einweisungen zum Frakturausschluss nach Sturz adressiert. In der Qualitätsinitiative „Interact“ (Interventions to Reduce Acute Care Transfers) hingegen werden gleich mehrere Tools in der Weiterbildung von Pflegefachkräften angewendet, die sowohl das frühzeitige Erkennen und Kommunizieren von Zustandsveränderungen als auch das vorausschauende Planen bei Bewohnern in palliativen Situationen beinhalten, allerdings konnte deren Wirksamkeit auf Krankenhauszuweisungen nicht nachgewiesen werden [62]. Heimarztmodelle ermöglichen neben dauerhafter Erreichbarkeit eine kontinuierliche interdisziplinäre Zusammenarbeit und gemeinsames Bahnen von Prozessen [64]. Allerdings beinhaltet der Wechsel der hausärztlichen Betreuung zu einem vulnerablen Zeitpunkt – dem Umzug in eine Pflegeeinrichtung – auch eine zumindest vorübergehende Einbuße durch fehlende Kenntnis der Bewohner:in [47].

Wenngleich viele Ansätze sinnvoll und in Deutschland unter Projektbedingungen auch erfolgreich erscheinen, sind für eine nachhaltige Implementierung die Möglichkeiten und Grenzen des Gesundheitssystems zu berücksichtigen. Für eine qualitativ gute Betreuung auch bei Zustandsveränderungen und in Notfallsituationen ist ausreichend vorhandenes und qualifiziertes Pflegefachpersonal eine Grundbedingung. Steht z. B. der Personalmangel im Vordergrund, so ist der zusätzliche Arbeitsaufwand durch die Versorgung akut erkrankter Bewohner:innen kaum leistbar – und auch mit Weiterbildungen ist vermutlich wenig zu erreichen. Werden Einweisungen dauerhaft vermieden, ist in den Einrichtungen ein erhöhter Pflegebedarf zu erwarten, dem Rechnung getragen werden muss.

Finanzielle Anreize zur Reduktion vermeidbarer Rettungsdiensteinsätze und Krankenhauszuweisungen von Pflegeheimbewohner:innen sind möglicherweise wirksam, ethisch jedoch umstritten. Eine notfallmedizinische Unterversorgung in einzelnen Einrichtungen kann mangels geeigneter Kontrollinstrumente nicht ausgeschlossen werden. Einen wichtigen Ansatzpunkt in Deutschland stellt die Entkoppelung der Vergütungsstruktur des Rettungsdienstes von der Transportleistung dar [65].

Methodische Herausforderungen in der Evaluation und wissenschaftlichen Bearbeitung dieses komplexen Themas zeigen sich darin, dass viele Untersuchungen u. a. zu „ambulant care sensitive conditions“ (ACSC) auf Diagnosen basieren, die erst nach der stationären Aufnahme gestellt wurden. Die initialen Geschehnisse oder Zustandsveränderungen bei Ersteinschätzung, die den Entscheidungsprozess zum weiteren Vorgehen steuern, wurden häufig nicht entsprechend dokumentiert und berücksichtigt [66]. Evidenz zu Notfallsituationen, die nicht zu Einweisungen geführt haben, ist kaum vorhanden.

Notwendig scheint zudem eine Analyse der Implementierung und Evaluation bisheriger Interventionen zur Reduzierung von Krankenhauszuweisungen aus Pflegeeinrichtungen, um aus Schwachstellen zu lernen und praktisch umsetzbare und erfolgreiche Maßnahmen zu entwickeln. Zu beachten sind bei allen Interventionen die zentrale Rolle des Pflegepersonals, die erforderlichen interprofessionellen Ansätze und – zukünftig zunehmend – digitalen Lösungen an den Schnittstellen der Notfallversorgung.

### Limitationen

Die hier angewandte Methode eines Scoping-Reviews in Anlehnung an Arksey und O’Malley (2005; [18]) stellt eine relativ einfache Herangehensweise dar, nicht alle methodologischen Erweiterungen nach Levac et al. (2010; [67]) und Peters et al. (2020; [19]) konnten vollständig umgesetzt werden. Beispielsweise wurden die Studienergebnisse kaum numerisch zusammengefasst. Das Screening der Publikationen wurde nicht durch 2 unabhängige Autor:innen durchgeführt, sondern erfolgte primär durch den Autor CB. Die weiteren Autor:innen wurden in diesen Prozess punktuell, jedoch nicht durchgehend einbezogen. Weitere Limitationen in der gewählten Suchstrategie des Scoping-Reviews sind u. a. die Beschränkung auf 2 Datenbanken bei insgesamt uneinheitlicher thematischer Verschlagwortung. Zudem wurde die Volltextsuche auf einen Zeitraum von 6 Jahren begrenzt. Die Trennschärfe der gebildeten Kategorien zur möglichen Systematisierung von Begleitumständen ist eingeschränkt, Mehrfachzuordnungen sind möglich. Darüber hinaus erschwert die vielfältige Methodik der eingeschlossenen Studien die Vergleichbarkeit der Ergebnisse. Die Übertragbarkeit von Interventionen aus dem internationalen Kontext ist aufgrund unterschiedlicher Gesundheitssystemvoraussetzungen ebenfalls begrenzt. Hinzu kommt, dass insbesondere aus vielen laufenden Forschungsprojekten in Deutschland weitere Ergebnisveröffentlichungen noch ausstehen und sich das Bild somit fortwährend verändert.

### Fazit

Das umfassende Verständnis der komplexen Versorgungsprozesse im Kontext von Notfallsituationen in Pflegeeinrichtungen ist eine wesentliche Grundlage für die Entwicklung und Implementierung wirklich effektiver Interventionen zur Minderung von Notfallsituationen mit nachfolgender Krankenhauszuweisung bei Pflegeheimbewohner:innen. Die Vorausplanung von Notfallsituationen, deren frühzeitige Erkennung und eine gelingende interprofessionelle Zusammenarbeit aller Beteiligten können zur adäquaten Versorgung in Notfallsituationen beitragen. Trotz zahlreicher bereits existierender Projekte und Ansätze sind zusätzliche systematische, prospektive Untersuchungen in deutschen stationären Pflegeeinrichtungen erforderlich, bei denen die Perspektive des Pflegepersonals zentrale Berücksichtigung finden sollte.

#### Infobox 1 Forschungslücken beim Thema Notfallsituationen in Pflegeeinrichtungen

- Anlässe für Krankenhauszuweisungen werden bislang fast nur aus medizinisch-diagnostischer Perspektive betrachtet
- Die Analyse erfolgt häufig retrospektiv
- Bestehende Begleitumstände sind bisher nicht umfassend beleuchtet
- Definitionen von „vermeidbaren“ Krankenhauszuweisungen sind uneinheitlich

## Supplementary Information



