# Supplementary material for: Emergency situations and emergency department visits in nursing homes—a scoping review about circumstances and healthcare interventions
Source: Bundesgesundheitsblatt Gesundheitsforschung Gesundheitsschutz. 2022 May 17;65(6):688–96. [Article in German] doi: 10.1007/s00103-022-03543-w (PMC9113071; doi:10.1007/s00103-022-03543-w)
Supplement: Supplementary file 1 [file 103_2022_3543_MOESM1_ESM.pdf]

Bundesgesundheitsblatt – Gesundheitsforschung – Gesundheitsschutz, Ausgabe 6/2022

Onlinematerial zum Beitrag:

Notfallsituationen und Krankenhauszuweisungen in Pflegeeinrichtungen – ein Scoping Review zu Begleitumständen und versorgungsrelevanten Maßnahmen

Carsten Bretschneider[1]*, Juliane Poeck[1]*, Antje Freytag[1], Andreas Günther[2], Nils Schneider[3], Sven Schwabe[3], Jutta Bleidorn[1]

*Die Autor:innen CB und JP trugen gleichermaßen zu dieser Arbeit bei

[1]Institut für Allgemeinmedizin, Universitätsklinikum Jena, Jena, Deutschland

[2]Fachbereich Feuerwehr, Braunschweig, Deutschland

[3]Institut für Allgemeinmedizin und Palliativmedizin, Medizinische Hochschule Hannover, Hannover, Deutschland

Korrespondenzadresse

Juliane Poeck M.Sc.

Universitätsklinikum Jena

Institut für Allgemeinmedizin

Bachstraße 18

07743 Jena

Deutschland

juliane.poeck@med.uni-jena.de

Inhalt:

Suchsyntax für die Artikelrecherche in den Datenbanken PubMed und CINAHL

Suchsyntax für die Artikelrecherche in den Datenbanken PubMed und CINAHL

("Nursing Homes"[Mesh] OR "Homes for the Aged"[Mesh]) AND ("Emergency Medicine"[Mesh] OR "Emergency Treatment"[Mesh] OR "Emergencies"[Mesh] OR emergenc*[Title/Abstract] OR patient Transfer*[Title/Abstract] OR "Patient Transfer"[Mesh] OR hospital admission*[Title/Abstract] OR patient admission*[Title/Abstract] OR "Advance Directives"[Mesh] OR "Emergency Medical Services"[Mesh] OR "Patient Readmission"[Mesh] OR avoidable hospital*[Title/Abstract] OR "Patient Admission"[Mesh] OR unnecessary transfer*[Title/Abstract] OR unnecessary hospital*[Title/Abstract] OR acute illness*[Title/Abstract])
